# Supplementary material for: Single-shot ultrafast imaging attaining 70 trillion frames per second
Source: Nat Commun. 2020 Apr 29;11:2091. doi: 10.1038/s41467-020-15745-4 (PMC7190645; doi:10.1038/s41467-020-15745-4)
Supplement: Supplementary file 1 — Supplementary Information [file 41467_2020_15745_MOESM1_ESM.pdf]

Supplementary Information

Single-shot ultrafast imaging attaining 70 trillion frames per second

Peng Wang[1], Jinyang Liang[1,2], and Lihong V. Wang[1,*]

[1] Caltech Optical Imaging Laboratory, Andrew and Peggy Cherng Department of Medical Engineering, Department of Electrical Engineering, California Institute of Technology, 1200 East California Boulevard, Mail Code 138-78, Pasadena, CA 91125, USA

[2] Present address: Centre Énergie Matériaux Télécommunications, Institut National de la Recherche Scientifique, 1650 boulevard Lionel-Boulet, Varennes, QC J3X1S2, CANADA

*Corresponding author: LVW@caltech.edu

Supplementary Note 1: Characterizations of the streak camera

Spectral sensitivity

Spectral sensitivity of the streak camera is taken into account in data acquisition. The measured quantum efficiency $Q_{\mathrm{S}}(\lambda)$ of the photocathode—the photon-sensitive element in the streak tube—is plotted in Supplementary Figure 2a. This data was provided by the streak camera's manufacturer. Note that the imaging optics and the input optics of the streak camera are assumed to have uniform transmission within each band. Since photoelectrons lose their wavelength fingerprints after generation and acceleration, the spectral sensitivity of the phosphor screen and the internal CCD camera are excluded.

Space-charge effect

The space-charge effect occurs when too many photoelectrons, confined at the focus of an electron imaging system, repel each other, limiting both spatial and temporal resolutions of the streak camera [1]. We studied the space-charge induced spread in the orthogonal directions at different optical intensities deposited at the entrance. A femtosecond laser with a 1-kHz pulse repetition rate was used as the source. Spread in the vertical direction y_{s} is equivalent to the spread in the time domain. As shown in Supplementary Figure 2b, at low intensity, the space-charge effect is negligible, but it becomes evident and intolerable quickly as the incident intensity increases. In practice, it is critical to control the incident intensity to minimize the space-charge effect (less than 2 pixels). On the other hand, the intensity has to be high enough to optimize the signal-to-noise ratio (SNR).

Response linearity

The data acquisition model of CUSP is based on the presumption that the streak camera responds linearly to the incident light intensity. Supplementary Figure 2c plots the measured response curve, exhibiting good linearity (>0.999) between the sensor pixel value and the incident light intensity. Only light intensities before the space-charge effect becomes evident were measured.

When characterizing the space-charge effect and measuring the response linearity, we illuminated the DMD with a uniform beam from the femtosecond laser. The DMD displayed a static pattern (see the insets in Supplementary Figure 2b) and the reflected light from the DMD is

relayed to the streak camera by the same optics in the CUSP system. No grating is present before the streak camera. We acquired images while varying the intensity of the illumination beam.

Other characteristics

The streak camera in this work has a tested sweeping linearity (i.e. linearity of the ultrafast sweeping voltage applied inside the streak tube) better than 0.996, which is sufficient to grant the data acquisition model in Supplementary Note 4 valid. In addition, at 10 THz sweeping speed and low light intensity, this streak camera has a tested temporal resolution of 230 fs [2]. However, this value is for 1D ultrafast imaging only. At this low light level (i.e. 1000 photons per pixel in the streak camera's raw image), the SNR would be too poor to produce clear CUSP images in single shot [2]. At the light level (i.e. 20000 photons per pixel in the streak camera's raw image) with a moderate SNR, the temporal resolution is typically larger than 400 fs [2, 3]. Fortunately, the temporal resolution of active CUSP (240 fs in Fig. 2c) is no longer bounded by this limit.

Supplementary Note 2: Details of the imaging section of the CUSP system

Tunable bandpass filter

As CUSP exploits spectral dispersion, it is important to characterize its performance at different wavelengths within the band of interest. For the active mode, we built a tunable bandpass filter immediately after the femtosecond laser (Supplementary Figure 3). As the grating (G) rotates, the pinhole selects different narrowbands with a full width at half maximum (FWHM) of 0.40 nm. The linearity between the selected wavelength and the rotation angle is >0.999.

Dispersion characterization

To study the dispersion characteristics of the active CUSP system, a small square was illuminated by both broadband femtosecond pulses and narrowband pulses at a wavelength varied by the tunable bandpass filter. The corresponding images were taken by the streak camera in focus mode. As plotted in Supplementary Figure 4b, the images shift in the horizontal direction linearly with respect to wavelength, matching well with the theoretical estimation based on the grating equation:

$$dx_{\mathrm{s}} = l d\theta = \frac{l}{\Lambda \cos\theta}\bigg|_{\theta=\theta_{\mathrm{c}}} d\lambda = \frac{l}{\Lambda\sqrt{1-(\lambda/\Lambda)^2}}\bigg|_{\lambda=\lambda_{\mathrm{c}}} d\lambda\,, \tag{1}$$

where dx_{s} is the lateral shift; l is the physical distance between the diffraction grating and the streak camera entrance; Λ is the grating period; λ_{c} is the center wavelength of the broadband pulse; θ_{c} is the first-order diffraction angle for λ_{c}. In this system, we have $l = 77$ mm, $\lambda_{\mathrm{c}} = 800$ nm and $\Lambda = 3.333$ µm, leading to a linear dispersion parameter of $\mu = dx/d\lambda = 23.7$ µm nm^{-1}. The experimental measurement has a linearity better than 0.999.

According to the diffraction theory, the spectral resolution of a grating is limited to $\lambda_{\mathrm{c}}/(\phi/\Lambda)$, where ϕ is the beam width on the grating (i.e., ϕ/Λ is the number of grating periods covered by the beam). In 70-Tfps active CUSP, a spectral resolution of 4.5 nm at $\lambda_{\mathrm{c}} = 800$ nm is desired, corresponding to a minimum $\phi_{\mathrm{min}} = 0.6$ mm, which is satisfied in practice. A similar dispersion characterization experiment for passive CUSP is shown in Supplementary Figure 13c.

<u>Effect of grating on image quality</u>

To test whether the introduction of the diffraction grating affects the image quality, we captured two groups of images, when the object was illuminated by various narrowband pulses from the tunable bandpass filter, as shown in Supplementary Figure 4c. In comparison to the images taken without the grating, there is negligible degradation in image quality. Meanwhile, the laterally shifted images are spectrally invariant, which serves as a foundation for the data acquisition model. A study on the passive CUSP system reached the same conclusion.

<u>Image distortion</u>

To calibrate for image distortion in the s-View with respect to the u-View, we first took images of a calibration target made of several dots in both views. Distortion was then quantified by locating the paired dots in the images (red crosses in Supplementary Figure 4d) and then computing a projective transformation $\boldsymbol{D}$ between them. Defined in Supplementary Equation 2, $\boldsymbol{D}$ includes anisotropic magnification (f_x and f_y), skew (s), displacement (c_x and c_y) and rotation (θ).

$$\boldsymbol{D} = \begin{bmatrix} f_x & 0 & 0 \\ s & f_y & 0 \\ c_x & c_y & 1 \end{bmatrix} \times \begin{bmatrix} \cos\theta & \sin\theta & 0 \\ -\sin\theta & \cos\theta & 0 \\ 0 & 0 & 1 \end{bmatrix}. \tag{2}$$

Supplementary Equation 2 was derived from the standard camera calibration matrix used in photography [4, 5]. A spatial point $(x_\mathrm{u}, y_\mathrm{u})$ in u-View is projected to $(x_\mathrm{s}, y_\mathrm{s})$ in s-View by

$$[x_\mathrm{s} \quad y_\mathrm{s} \quad 1] = [x_\mathrm{u} \quad y_\mathrm{u} \quad 1] \times \boldsymbol{D}\,. \tag{3}$$

Supplementary Note 3: Details of the illumination section of the active CUSP system

Frequency-resolved optical gating (FROG) measurement of the femtosecond pulse

The original femtosecond pulse before entering the CUSP system was characterized by a commercial FROG instrument (Swamp Optics, GRENOUILLE 8-20-377-USB), plotted in Supplementary Figures 5a-5d. In Supplementary Figures 5c and 5d, we converted phase φ to optical frequency difference and wavelength. The original femtosecond pulse has an intensity FWHM of 48 fs, a minimum temporal chirp, and a pulse front tilt of less than 20 fs over the whole beam. Since optical components introduce group delay dispersion, we also measured the pulse after it traveled through the entire imaging section (Supplementary Figures 5f-5h). The pulse was linearly chirped to 120 fs in FWHM. It is reasonable to ignore this 72 fs elongation since it is beyond the resolving power of active CUSP.

Intensity auto-correlator for stretched pulse characterization

Active CUSP depends on accurate duration measurements of the optical pulses chirped by glass rods of various lengths. Therefore, we built an intensity auto-correlator, whose schematic is shown in Supplementary Figure 5i. The detected sum-frequency-generation (SFG) signal is proportional to the auto-correlation of the input pulse intensity. To scan the time delay, two retro-reflection mirrors (pink dashed box in Supplementary Figure 5i) are mounted on a motorized stage with a 300 nm step size (equivalently 2 fs in time delay).

Pulse train generation

The schematic of a pair of beamsplitters in proximity is given in Supplementary Figure 6a. They operate in non-resonance mode. The time delay between neighboring sub-pulses is determined by the physical gap h_b:

$$t_\mathrm{sp} = \frac{2h_\mathrm{b}}{c} n_0 \, . \tag{4}$$

Here, $n_0 = 1$ in air, and c is the speed of light. A high-precision micrometer stage controls the position of one beamsplitter. Note that the coated sides face the cavity so that all the sub-pulses go through the beamsplitter substrates the same number of times, which is also the minimum number of times. To protect the laser cavity from unwanted back-reflection, they are slightly tilted. Note that for $t_\mathrm{sp} = 2$ ps in our experiments, $2h_\mathrm{b} = 600$ μm, far below the measured coherence length of 20 μm, therefore there is no interference between the neighboring sub-pulses.

We experimentally measured the intensity of each sub-pulse by the streak camera at 10 THz sweeping speed. Beamsplitters of higher reflectivity generate more usable sub-pulses with a penalty of reduced pulse energy. Supplementary Figures 6b and 6c show the results of generating five and seven usable sub-pulses, respectively. Due to the response linearity of the streak camera (Supplementary Figure 2c), the normalized pixel value equals the normalized light intensity. As the sub-pulse order p increases, its intensity falls exponentially. Therefore, only the first few were used, while the rest were discarded due to the lower intensity, which would lead to lower SNR.

<u>Pulse stretching by glass rods</u>

Glass rods of various lengths, made of N-SF11, were employed to linearly chirp and stretch the femtosecond pulse to picosecond long. N-SF11 has a group velocity dispersion (GVD) of 187.5 $\mathrm{fs}^2\ \mathrm{mm}^{-1}$ at $\lambda_\mathrm{c} = 800$ nm, which is translated to a GVD parameter of $D_\lambda = -0.555$ fs mm nm^{-1} by

$$D_\lambda = -\frac{2\pi c}{\lambda_\mathrm{c}^2} \cdot \mathrm{GVD} \, . \tag{5}$$

For a bandwidth of 38 nm, a 270-mm-long N-SF11 rod and a 95-mm-long one stretch the 48-fs pulse to 5.7 ps and 2.0 ps, corresponding to negative chirp parameters of $\eta_\mathrm{rod\_1} = -150$ fs nm^{-1} and $\eta_\mathrm{rod\_2} = -52.7$ fs nm^{-1}, respectively. The 270-mm rod and the 95-mm rod were deployed for the experiments shown in Fig. 2 and Fig. 3, respectively.

Supplementary Figure 7 gives the measurements of the pulse before and after being stretched. As in Supplementary Figure 2a, the grating disperses a square-shaped object in the horizontal direction. When there is no rod, the time-sheared image acquired in streak mode resembles that acquired in focus mode. However, a large temporal chirp becomes evident when a stretching rod is inserted. The measured time delay between the shortest (785 nm) and the longest (823 nm) wavelengths matched well with the theoretical calculations above. Additionally, the temporal chirp by a homogeneous isotropic rod can be treated as a linear one. This linearity is demonstrated by the centers of the light intensity distributions at different wavelengths, matching well with the theoretical estimation based on a linear temporal chirp (green solid lines in Supplementary Figures 7c-7e). In Supplementary Figures 7f and 7g, the measurements from the auto-correlator and spectrometer also confirm the stretch. After converting the wavelength to time through the linear chirp parameter, the spectrum (red short-dashed line) yields an intensity profile well matching the Gaussian-pulse approximation (black long-dashed line) derived from the auto-correlator signals.

Supplementary Note 4: Details of CUSP's data acquisition

Data acquisition of passive-mode CUSP

To begin with, we present the detailed data acquisition process of passive CUSP (Supplementary Figure 8). To simplify the explicit expressions for both u-View and s-View, we make the following assumptions, without loss of generality. First, the entire imaging system has a magnification of 1×. Second, the DMD, the external CCD camera, the internal CCD of the streak camera have the same pixel size, denoted as d, and their pixels are matched. Third, the scene is perfectly imaged to the DMD. To simplify the notations, we choose a voxel of (d, d, τ, δ), in the x-y-t-λ space, where $\tau = d/v$ and $\delta = d/|\mu|$. Here v is the temporal shearing speed of the streak camera and μ is the spectral dispersion parameter.

In u-View, the dynamic scene $I(x, y, t, \lambda)$ is imaged on the external CCD camera through both low-pass filtering caused by the optical components, denoted as $\boldsymbol{F}_{\mathbf{u}}$, and spatiotemporal-spectrotemporal integration, denoted as $\boldsymbol{T}$,

$$I_{F_{\mathrm{u}}}(x_{\mathrm{u}}, y_{\mathrm{u}}, t, \lambda) = \boldsymbol{F}_{\mathbf{u}}\{I(x, y, t, \lambda)\}\,, \qquad (6)$$

$$E_{\mathrm{u}}[m,n] = \boldsymbol{T}\{Q_{\mathrm{u}}(\lambda) \cdot I_{F_{\mathrm{u}}}(x_{\mathrm{u}}, y_{\mathrm{u}}, t, \lambda)\} \tag{7}$$

$$= \int dx_{\mathrm{u}} \int dy_{\mathrm{u}} \left\{ \left[\int dt \int d\lambda \; Q_{\mathrm{u}}(\lambda) \cdot I_{F_{\mathrm{u}}}(x_{\mathrm{u}}, y_{\mathrm{u}}, t, \lambda) \right] \cdot rect\left[\frac{x_{\mathrm{u}}}{d} - \left(m + \frac{1}{2}\right), \frac{y_{\mathrm{u}}}{d} - \left(n + \frac{1}{2}\right) \right] \right\}.$$

In Supplementary Equation 6, x_{u} and y_{u} are the spatial coordinates of the external CCD camera. In Supplementary Equation 7, $E_{\mathrm{u}}[m,n]$ represents the optical energy measured by the $[m,n]$ pixel on the CCD, and $Q_{\mathrm{u}}(\lambda)$ is the quantum efficiency of the external CCD camera. Here, we use the 2D rectangular function [6] "$rect$" to represent one camera's physical pixel, which is located at $[m,n]$ and has a lateral dimension of $d \times d$.

In s-View, we firstly apply spatial encoding to $I(x,y,t,\lambda)$ by a pseudo-random binary pattern $C(x,y)$ displayed on the DMD, giving the following intensity distribution:

$$I_{\mathrm{C}}(x,y,t,\lambda) = C(x,y)I(x,y,t,\lambda)\,. \tag{8}$$

The encoded scene is then relayed to the entrance port of the streak camera by passing through the imaging system, which also introduces spatial low-pass filtering $\boldsymbol{F}_{\mathbf{s}}$:

$$I_{F_{\mathrm{s}}}(x,y,t,\lambda) = \boldsymbol{F}_{\mathbf{s}}\{I_{\mathrm{C}}(x,y,t,\lambda)\}\,. \tag{9}$$

Next, an image distortion operator of the s-View is applied:

$$I_{\mathrm{D}}(x,y,t,\lambda) = \boldsymbol{D}\left\{I_{F_{\mathrm{s}}}(x,y,t,\lambda)\right\}. \tag{10}$$

In the next step, the dynamic scene is spectrally dispersed by the diffraction grating. Here, we define an intermediate coordinate system right at the entrance port of the streak camera: $x' = x + \mu(\lambda - \lambda_0)$, $y' = y$. Hence, the dispersed image I_{S_λ} is given by

$$I_{S_\lambda}(x',y',t,\lambda) = \boldsymbol{S}_{\boldsymbol{\lambda}}\{I_{\mathrm{D}}(x,y,t,\lambda)\} = I_{\mathrm{D}}(x' - \mu(\lambda - \lambda_0), y', t, \lambda)\,. \tag{11}$$

Afterward, the dispersed scene is captured by the streak camera. Here, the quantum efficiency $Q_{\mathrm{s}}(\lambda)$ of the streak camera photocathode kicks in so that the generated photoelectron energy is

$$I_{\mathrm{phe}}(x',y',t,\lambda) = Q_{\mathrm{s}}(\lambda) \cdot I_{S_\lambda}(x',y',t,\lambda)\,. \tag{12}$$

Here, the subscript "phe" stands for "photoelectrons". We define the spatial axes of the streak camera as $x_{\mathrm{s}} = x'$ and $y_{\mathrm{s}} = y' + vt$. Thus, the temporal shearing along the vertical spatial axis can be expressed by

$$I_{S_t}(x_\mathrm{s}, y_\mathrm{s}, t, \lambda) = \boldsymbol{S_t}\{I_\mathrm{phe}(x', y', t, \lambda)\} = Q_\mathrm{s}(\lambda) \cdot I_{S_\lambda}(x_\mathrm{s}, y_\mathrm{s} - vt, t, \lambda) \,. \tag{13}$$

Finally, $I_{S_t}(x_\mathrm{s}, y_\mathrm{s}, t, \lambda)$ is imaged to an internal CCD sensor by spatiotemporal-spectrotemporal integration $\boldsymbol{T}$. Akin to the *u*-View, the optical energy measured by the $[m, n]$ pixel on the CCD takes the form

$$E_\mathrm{s}[m, n] = \boldsymbol{T}\{I_{S_t}(x_\mathrm{s}, y_\mathrm{s}, t, \lambda)\} \tag{14}$$

$$= \int dx \int dy \left\{ \left[\int dt\, I_{S_t}(x_\mathrm{s}, y_\mathrm{s}, t, \lambda) \right] \cdot rect\left[\frac{x_\mathrm{s}}{d} - \left(m + \frac{1}{2}\right), \frac{y_\mathrm{s}}{d} - \left(n + \frac{1}{2}\right) \right] \right\}.$$

Taking Supplementary Equations 11-13 into Supplementary Equation 14, we get

$$\begin{aligned} E_\mathrm{s}[m, n] = \int dx \int dy \Bigg\{ \bigg[\int dt \int d\lambda\, Q_\mathrm{s}(\lambda) \cdot I_\mathrm{D}(x_\mathrm{s} - \mu(\lambda - \lambda_0), y_\mathrm{s} - vt, t, \lambda) \bigg] \\ \cdot\, rect\left[\frac{x_\mathrm{s}}{d} - \left(m + \frac{1}{2}\right), \frac{y_\mathrm{s}}{d} - \left(n + \frac{1}{2}\right) \right] \Bigg\}. \end{aligned} \tag{15}$$

The image pixel value that is read out from the streak camera is linearly proportional to the deposited optical energy E_s (see Supplementary Figure 2c).

To use this model in a compressed sensing-based reconstruction algorithm, it is required to derive a discrete-to-discrete model by discretizing the dynamic scene:

$$\begin{aligned} I[m, n, k, q] = \int d\lambda \int dt \int dx \int dy\, I(x, y, t, \lambda) \\ \cdot\, rect\left[\frac{x}{d} - \left(m + \frac{1}{2}\right), \frac{y}{d} - \left(n + \frac{1}{2}\right), \frac{t}{\tau} - \left(k + \frac{1}{2}\right), \frac{\lambda - \lambda_0}{\delta} - \left(q + \frac{1}{2}\right) \right]. \end{aligned} \tag{16}$$

In Supplementary Equation 16, m, n, k, q are non-negative integers. Therefore, the measurement of the *u*-View can be approximated by

$$E_\mathrm{u}[m, n] = \frac{d^4}{v|\mu|} \sum_k \sum_q Q_\mathrm{u}[q] \cdot (h_\mathrm{u} * I)[m, n, k, q] \,. \tag{17}$$

Here, h_u is the discrete convolution kernel of the operator $\boldsymbol{F}_\mathbf{u}$, and * stands for the discrete 2D spatial convolution operation.

For the *s*-View, the encoding mask is discretized to

$$C[m,n] = \int dx \int dy\, C(x,y) \cdot rect\left[\frac{x}{d} - \left(m + \frac{1}{2}\right), \frac{y}{d} - \left(n + \frac{1}{2}\right)\right]. \quad (18)$$

Then, the encoded scene becomes

$$I_{\mathrm{C}}[m,n,k,q] = C[m,n] \cdot I[m,n,k,q]\,. \quad (19)$$

Eventually, the discretized form of the streak camera measurement is represented by

$$E_{\mathrm{s}}[m,n] = \frac{d^4}{v|\mu|} \sum_k \sum_q Q_{\mathrm{s}}[q] \cdot (h_{\mathrm{s}} * I_{\mathrm{C}})[m_{\mathrm{D}} - q, n_{\mathrm{D}} - k, k, q]\,, \quad (20)$$

where h_{s} is the discrete convolution kernel of the operator $\boldsymbol{F_{\mathrm{s}}}$, m_{D} and n_{D} are the discrete coordinates transformed according to the distortion operator $\boldsymbol{D}$ [see Supplementary Equation 3]. Note that the entire derivation process from Supplementary Equation 6 to Supplementary Equation 20, computing E_{u} and E_{s} from I, has considered all the operations in the joint operator $\boldsymbol{O}$ in Equation (1) in Methods.

<u>Data acquisition of active-mode CUSP</u>

In the passive version of CUSP, time and spectrum are independent, therefore we can directly apply the general model derived above. However, in active mode, spectrum and time are dependent because we use the spectrum for time stamping. Consequently, the general model has to be modified. To start with, the dynamic scene, illuminated by a train of chirped pulses, can be expressed by

$$I(x,y,t) = I\left(x, y, pt_{\mathrm{sp}} + \eta(\lambda - \lambda_0)\right) = I\big(x, y, t(p,\lambda)\big)\,. \quad (21)$$

All the parameters were defined in Main Text. Note that we can still use Supplementary Equation 16 as its discrete form, however, $k = round\big(pt_{\mathrm{sp}}/\tau\big)$ is a non-negative integer that is assigned to the sub-pulse sequence p only.

For the u-View, Supplementary Equations 6 and 7 are replaced by

$$I_{F_{\mathrm{u}}}\big(x_{\mathrm{u}}, y_{\mathrm{u}}, t(p,\lambda)\big) = \boldsymbol{F_{\mathrm{u}}}\big\{I\big(x, y, t(p,\lambda)\big)\big\}\,, \quad (22)$$

$$E_{\mathrm{u}}[m,n] = \boldsymbol{T}\big\{Q_{\mathrm{u}}(\lambda) \cdot I_{F_{\mathrm{u}}}\big(x_{\mathrm{u}}, y_{\mathrm{u}}, t(p,\lambda)\big)\big\} \quad (23)$$

$$= \int dx_{\mathrm{u}} \int dy_{\mathrm{u}} \left\{ \left[\sum_p \int d\lambda\ Q_{\mathrm{u}}(\lambda) \cdot I_{F_{\mathrm{u}}}\big(x_{\mathrm{u}}, y_{\mathrm{u}}, t(p,\lambda)\big) \right] \cdot rect \left[\frac{x_{\mathrm{u}}}{d} - \left(m + \frac{1}{2}\right), \frac{y_{\mathrm{u}}}{d} - \left(n + \frac{1}{2}\right) \right] \right\}.$$

Therefore, the discrete-to-discrete model for this view can be adapted from Supplementary Equation 17:

$$E_{\mathrm{u}}[m,n] = \frac{d^4}{v|\mu|} \sum_p \sum_q Q_{\mathrm{u}}[q] \cdot (h_{\mathrm{u}} * I)[m,n,k(p),q]\ , \tag{24}$$

where $k = round\big(pt_{\mathrm{sp}}/\tau\big)$, $p = 0,1,2,\dots,(P-1)$, and $q = 0,1,2,\dots,\big((N_{\mathrm{ta}}/P) - 1\big)$. N_{ta} is the number of recorded frames in the active mode, defined in Main Text.

For the s-View, we can basically follow the same derivation process from Supplementary Equation 8 to Supplementary Equation 11, but replace t by pt_{sp} and re-define the vertical axis of the streak camera as $y_{\mathrm{s}} = y' + v\left(pt_{\mathrm{sp}} + \eta(\lambda - \lambda_0)\right)$. As a result, the optical energy received by the internal CCD is

$$E_{\mathrm{s}}[m,n] \tag{25}$$

$$= \int dx \int dy \left\{ \left[\sum_p \int d\lambda\, Q_{\mathrm{u}}(\lambda) \cdot I_{\mathrm{D}}\left(x_{\mathrm{s}} - \mu(\lambda - \lambda_0), y_{\mathrm{s}} - v\left(pt_{\mathrm{sp}} + \eta(\lambda - \lambda_0)\right), t(p,\lambda)\right) \right] \cdot rect\left[\frac{x_{\mathrm{s}}}{d} - \left(m + \frac{1}{2}\right), \frac{y_{\mathrm{s}}}{d} - \left(n + \frac{1}{2}\right) \right] \right\}.$$

Similarly, its discrete-to-discrete model is given by

$$E_{\mathrm{s}}[m,n] = \frac{d^4}{v|\mu|} \sum_p \sum_q Q_{\mathrm{s}}[q] \cdot (h_{\mathrm{s}} * I_{\mathrm{C}})[m_{\mathrm{D}} - q, n_{\mathrm{D}} - r(p,q), k(p), q]\ , \tag{26}$$

in which $r = round\left[\left(pt_{\mathrm{sp}} + \eta(\lambda - \lambda_0)\right)/\tau\right] = round\left[\left(pt_{\mathrm{sp}} + \eta q\delta\right)/\tau\right]$, $k = round\big(pt_{\mathrm{sp}}/\tau\big)$, $p = 0,1,2,\dots,(P-1)$, and $q = 0,1,2,\dots,\big((N_{\mathrm{ta}}/P) - 1\big)$.

Supplementary Note 5: Details of CUSP's image reconstruction

In the TwIST algorithm, the regularization parameter ξ has values of 0.6, 0.5 and 1.0 for the three sets of experiments shown in Figs. 2-4, respectively. They were chosen via trial-and-error while the results were inspected. The maximum number of iterations was 50. The total variation defined by Supplementary Equation 27 was selected as the regularizer,

$$\begin{aligned}\Phi(I) &= \Phi_{\mathrm{TV}}(I)\\ &= \sum_{k}\sum_{q}\sum_{m,n}\sqrt{(I[m,n+1,k,q]-I[m,n,k,q])^2+(I[m+1,n,k,q]-I[m,n,k,q])^2}\\ &+\sum_{m}\sum_{n}\sum_{k,q}\sqrt{(I[m,n,k+1,q]-I[m,n,k,q])^2+(I[m,n,k,q+1]-I[m,n,k,q])^2}\,.\end{aligned} \tag{27}$$

To implement the CUSP reconstruction, it is required to accurately measure spatial low-pass filtering operators $\boldsymbol{F}_{\mathbf{u}}$ and $\boldsymbol{F}_{\mathbf{s}}$, the encoding matrix $C[m,n]$, the distortion matrix $\boldsymbol{D}$, and the adjoint of operator $\boldsymbol{O}$ in Methods. Supplementary Note 2 details the experimental calibration of $\boldsymbol{D}$. The measurements of the other operators were described in a previous publication [7].

In the experiments in Fig. 3, the reconstructed intensity is the transmittance of the Kerr gate T_{Kerr}, multiplied by both the spectrum of the illumination $\Psi_{\mathrm{illum}}(\lambda)$ (see the green plot in Fig. 2d) and the relative sub-pulse intensity I_p (see Supplementary Figure 6). Therefore, the reconstructed intensity has to be divided by both $\Psi_{\mathrm{illum}}(\lambda)$ and I_p in order to get the transmittance dynamics in Supplementary Movie 2 and Fig. 3.

Supplementary Note 6: Additional data for imaging a linear optical phenomenon

Spatial and temporal chirps can be simultaneously generated by a pair of gratings of the same grating period Λ_{gp} facing each other, as illustrated in Supplementary Figure 9a. The two gratings were spaced by a distance of h_{gp} in the direction perpendicular to the grating surface. Assuming the incident angle α and the first-order diffraction angle γ_{c} of the center wavelength λ_{c}, the spatial chirp of the output beam in the x direction can be derived by the geometric relation

$$dx = \frac{h_{\mathrm{gp}} \cos\alpha}{(\cos\gamma)^2}\bigg|_{\gamma=\gamma_{\mathrm{c}}} d\gamma\,. \tag{28}$$

Based on the grating equation, we get

$$d\gamma = \frac{d\lambda}{\Lambda_{\mathrm{gp}} \cos\gamma}\bigg|_{\gamma=\gamma_{\mathrm{c}}}. \tag{29}$$

Substituting Supplementary Equation 28 into Supplementary Equation 29, we have the expression for spatial chirp:

$$dx = \frac{h_{\mathrm{gp}} \cos\alpha}{\Lambda_{\mathrm{gp}}(\cos\gamma)^3}\bigg|_{\gamma=\gamma_{\mathrm{c}}} d\lambda\,. \tag{30}$$

In the experimental setup, $h_{\mathrm{gp}} = 298$ mm, $\alpha = 27.8°$ and $\gamma_{\mathrm{c}} = 45°$, giving a linear spatial chirp of $\varepsilon = dx/d\lambda = 0.224$ mm nm^{-1}.

Temporal chirp is defined as the difference in time of arrival between neighboring wavelengths in the xy plane. The optical path length at any wavelength λ is

$$l_{\mathrm{gp}} = l_{\mathrm{gp-a}} + l_{\mathrm{gp-b}} = \frac{h_{\mathrm{gp}}}{\cos\gamma} + \frac{h_{\mathrm{gp}} \cos(\alpha+\gamma)}{\cos\gamma}\,. \tag{31}$$

Taking the derivative of Supplementary Equation 31 gives

$$dl_{\mathrm{gp}} = \frac{h_{\mathrm{gp}}(\sin\gamma - \sin\alpha)}{(\cos\gamma)^2}\bigg|_{\gamma=\gamma_0} d\gamma\,. \tag{32}$$

Considering the speed of light in air c, the difference in time of arrival is

$$dt_{\mathrm{gp}} = \frac{dl_{\mathrm{gp}}}{c} = \frac{h_{\mathrm{gp}}\lambda}{c\Lambda_{\mathrm{gp}}^2(\cos\gamma)^3}\bigg|_{\substack{\gamma=\gamma_{\mathrm{c}}\\ \lambda=\lambda_{\mathrm{c}}}} d\lambda\,. \tag{33}$$

The temporal chirp parameter is $\eta_{\mathrm{gp}} = dt_{\mathrm{gp}}/d\lambda = 202.6$ fs nm^{-1}. Note that the grating pair produces a positive temporal chirp, meaning that shorter wavelengths precede the longer wavelengths. The sign of η_{gp} is opposite to that from the 270-mm-long glass rods, as shown in Supplementary Note 3. As a result, the overall temporal chirp parameter for this experiment is $\eta = \eta_{\mathrm{rod\_1}} + \eta_{\mathrm{gp}} = 52.6$ fs nm^{-1}.

To directly measure the spatial chirp, in Supplementary Figure 9b, a fiber-coupled spectrometer scanned across the chirped beam along the x direction and the measured peak wavelengths are plotted against the detector's physical position.

For the temporal chirp measurement, in Supplementary Figure 9c, the beam firstly illuminated a 1D narrow slit in the object plan, and the streak camera recorded the image at 10 THz sweeping speed. The diffraction grating in front of the streak camera was not in place for this measurement. Note that the horizontal axis in the streak image is converted to wavelength based on the spatial chirp relation found in Supplementary Figure 9b.

Supplementary Note 7: Implementation of T-CUP

We compared CUSP and the state-of-the-art T-CUP technique in Main Text. Switching from active CUSP to T-CUP is achieved by removing the diffraction grating before the streak camera. However, because of the grating's diffraction angle, the mirror after the grating is no longer at the right position and the right angle for T-CUP. Our solution is to place the grating and the mirror for CUSP on a kinematic magnetic mount, and place another mirror on a second magnetic mount for T-CUP. Both mounts are coupled with a common magnetic base fixed to the optical table. Finally, the position of the streak camera also has to be adjusted in the direction of light propagation by a few millimeters. All the other components remain intact. This system design allows minimum changes in the setup and reliable transitions between CUSP and T-CUP. See Supplementary Figure 10 for the system's schematic. Note that the tested repeatability (20 μrad) of this kinematic magnetic mount is sufficient for our system. We used T-CUP's maximum imaging speed of 10 Tfps, which is limited by the sweeping speed of the fastest streak camera [3, 8].

Supplementary Note 8: Additional data for imaging a nonlinear optical phenomenon

<u>Experimental setup</u>

Supplementary Figure 11 gives the detailed schematic of the Kerr gate setup. The original 48-fs pulse is horizontally polarized (x). It is split into the gate and detection arms by a beamsplitter of minimal GVD. In the gate arm, a hollow-roof prism mirror, mounted on a high-precision

motorized stage of 300 nm step size (equivalently 2 fs in time delay), tunes the time delay between the two arms. A half-wave-plate (HWP1) rotates the polarization to vertical (y). Two cylindrical lenses of different focal lengths reshape the round beam into an elliptical form and focus it into the BGO crystal. The short-focal-length lens (CyL2) has motion control along the x direction to move the focal spot in and out of the field of view (Experiments 1 and 2 in Fig. 3). In the detection arm, which was coupled with the illumination section of the active CUSP system, the beam size is firstly shrunk by 2 times. Another half-wave-plate (HWP2) aligns the polarization angle of the detection light to that of the first polarizer's (P1) polarization axis. An N-SF11 rod of 95-mm long with a chirp parameter of $\eta = \eta_{\mathrm{rod\_2}} = -52.7$ fs nm^{-1} was deployed for 70-Tfps imaging.

BGO is known for producing multi-photon-absorption induced fluorescence (MPF) since its 4.8 eV bandgap is close to the three-photon energy of the 800 nm light [9]. We used a long-pass filter to eliminate this undesired fluorescence. The measured spectrum (inset of Supplementary Figure 11) proves that the long-pass filter with 715 nm cut-off can effectively block the MPF.

<u>Estimation of phase retardation</u>

Kerr effect introduces transient birefringence inside the medium [10]. In other words, the refractive index along the polarization direction of the gate light (y) is changed linearly proportionally to the gate pulse intensity,

$$\Delta n = \kappa \left|\vec{E}_{\mathrm{gate}}\right|^2 = \kappa I_{\mathrm{gate}} \,. \tag{34}$$

The nonlinearity coefficient κ is proportional to the third-order susceptibility $\chi^{(3)}$. As a result, the detection light accumulates different phases between two orthogonal polarizations where it meets the gate pulse in the BGO. Since P2's and P1's polarization axes are orthogonal to each other in the Kerr gate setup, the transmittance of the Kerr gate is

$$T_{\perp} = T_{\mathrm{Kerr}} = \frac{1 - cos\,\varphi}{2} \,. \tag{35}$$

Here, $\varphi = k_{\mathrm{BGO}} \Delta n l_{\mathrm{Kerr}}$, in which k_{BGO} is the angular wavenumber in BGO, and l_{Kerr} is the interaction length between the gate pulse and the detection pulse. When the detection light meets the traveling gate pulse, φ has a finite value, leading to high transmittance. When the detection misses the gate, $\varphi = 0$, displaying a dark background $T_{\perp} = 0$.

In order to measure the phase retardation φ, we rotated P2 to be aligned with P1. In this case, the transmittance after P2 becomes

$$T_{\parallel} = \frac{1 + cos\,\varphi}{2}. \tag{36}$$

We computed $\varphi = \pi/9$ near the focus of the gate pulse with a peak power density of 5.6×10^{14} mW cm^{-2}.

Stability of the Kerr gate

Taking the derivative of Supplementary Equation 35 and considering that φ is proportional to I_{gate}, we obtain the following relation:

$$\frac{dT_{\text{Kerr}}}{T_{\text{Kerr}}} = \frac{\varphi\, sin\,\varphi}{1 - cos\,\varphi} \cdot \frac{dI_{\text{gate}}}{I_{\text{gate}}}. \tag{37}$$

Therefore, the fractional change of the Kerr gate transmittance is proportional to the fractional change of the gate pulse intensity. We define coefficient $A = (\varphi \sin \varphi)/(1 - \cos \varphi)$, which is plotted in Supplementary Figure 12a. In our Kerr gate, the phase retardation φ ranges from 0 to $\pi/9$, where the transmittance is sensitive to random fluctuations in the gate pulse intensity.

In the experimental study, a total of 200 consecutive shots were captured while the time delay between the gate pulse and the detection pulse was fixed. Here, a single 48-fs pulse was used as the detection pulse. The transmittance profile varied dramatically with a relative change of 0.175 (standard deviation/mean, or SD/M for short) as shown in Supplementary Figure 12c. A set of reference images were also taken when the gate pulse was blocked and the polarizers P1 and P2 were set parallel. These reference images directly measure the laser intensity fluctuation, showing a relative change of only 0.016. As summarized in Supplementary Figure 12c, the Kerr gate transmittance has SD/M 11 times as large as that of the reference intensity.

Such a high sensitivity to random fluctuations calls for single-shot ultrafast imaging, and conventional pump-probe imaging may not be applicable. An experimental comparison is also shown in Supplementary Movie 3. In the pump-probe measurement, a 48-fs pulse was used as the probe, and the time delay was varied in a step size equal to the frame interval in active CUSP. The peak power density of the gate pulse was the same as that used in CUSP.

The locally normalized transmittance at a spatial position is plotted in Supplementary Movie 3. Compared with CUSP and T-CUP, the pump-probe method displays a much noisier transmittance evolution. In a pump-probe measurement, one image is acquired for a preset time delay. Therefore, 980 independent acquisitions were used to observe the entire dynamics in Fig. 3b. Since the fractional intensity fluctuation is 11 times than that in CUSP, averaging over 121 images per time delay is required to compensate for the fluctuation. Therefore, pump-probe imaging needs >10^5 laser shots to acquire the dynamics with the same stability and sequence depth as CUSP. In other words, CUSP outperforms pump-probe imaging by >10^5 times in data acquisition throughput.

Supplementary Note 9: Additional data for SR-FLIM

Experimental setup

The schematic of the entire passive CUSP system for SR-FLIM is shown in Supplementary Figure 13a. Fluorescence of short lifetime is known to have low quantum efficiency; therefore, it is critical to make maximal use of all the emitted photons. Different from the active CUSP system in Fig. 1, where the DMD was placed in retro-reflection, in this modified system, two separate sets of optical lenses were used to project the image onto the DMD and then relay the encoded image from the DMD to the streak camera, respectively. The 50/50 (R/T) beamsplitter was also replaced by a 90/10 (R/T) one. Thus, this scheme harnesses 3.6× photons in s-View, compared with that in Fig. 1.

Similar to the active CUSP system, a diffraction grating of period $\Lambda_{\mathrm{FLIM}} = 1.667$ μm was inserted at a distance $l_{\mathrm{FLIM}} = 14$ mm from the entrance port of the streak camera (Supplementary Figure 13b). Within the emission spectrum of Rhodamine 6G (Rh6G), a linear dispersion parameter of $\mu_{\mathrm{FLIM}} = -9\ \mu\mathrm{m\ nm}^{-1}$ was obtained based on Supplementary Equation 1.

Two-dimensional point spread function (PSF)

To study the temporal and spectral resolutions of passive CUSP for SR-FLIM at 0.5 Tfps, an object with no fluorescence was illuminated by a 532-nm 10-ps pulse. After CUSP reconstruction, we spatially integrated the reconstructed intensity and plotted it in the t-λ space (Supplementary

Figure 14a). It is the impulse response of the system, or in other words, the 2D PSF. The 1D PSFs in the spectral and time domains give FWHMs of 13 nm and 20 ps, respectively. A deconvolution from a 10-ps Gaussian pulse results in a 17-ps FWHM in the time domain.

Fluorescence lifetimes

There are two pathways for fluorophores to decay from the excited state to the ground state: radiative relaxation and non-radiative relaxation [11]. The first one, giving off photons, can be observed by optical microscopy. Relaxation does not happen instantaneously, but has a finite lifetime τ^{f} determined by the rates of radiative relaxation σ_{r} and non-radiative relaxation σ_{nr},

$$\sigma = \sigma_{\mathrm{r}} + \sigma_{\mathrm{nr}}\,, \tag{38}$$

$$\frac{1}{\tau^{\mathrm{f}}} = \frac{1}{\tau_{\mathrm{r}}{}^{\mathrm{f}}} + \frac{1}{\tau_{\mathrm{nr}}{}^{\mathrm{f}}}\,. \tag{39}$$

Lifetimes of commonly used fluorophores typically range from hundreds of picoseconds to tens of nanoseconds [12]. Since non-radiative decay does not emit photons, the fluorescence quantum efficiency can be expressed as $Q^{\mathrm{f}} = \sigma_{\mathrm{r}}/\sigma = \tau^{\mathrm{f}}/\tau_{\mathrm{r}}{}^{\mathrm{f}}$.

In our SR-FLIM experiment, a high fluorophore concentration leads to a shorter lifetime due to the formation of dimers and higher aggregates of the fluorophores [11], which contributes to more non-radiative pathways, equivalently shorter $\tau_{\mathrm{nr}}{}^{\mathrm{f}}$. At the same time, the radiative relaxation rate remains independent of concentration. As a result, the quantum efficiency Q^{f} also drops [13].

The fluorescence decay is typically expressed by the following equation:

$$U(t) = \begin{cases} ae^{-\frac{t}{\tau^{\mathrm{f}}}}, & t > 0 \\ 0, & t \le 0 \end{cases}, \tag{40}$$

which assumes that a single exponential term is sufficient to describe Rh6G's fluorescence decay behavior [11]. Fluorescence excitation is considered instantaneous. The acquired data in the time domain is a convolution of Supplementary Equation 40 and the system's 1D PSF in the time domain. Nonlinear least squares fitting (MATLAB R2017b) was applied to retrieve the lifetime. The fluorescence decay curves at peak wavelengths and their exponential fits are summarized in Supplementary Figure 14b.

Standard lifetime measurement

Lifetime measurements made by CUSP and a standard method were quantitatively compared. In the standard measurement, we directly imaged uniform fluorescence samples using the streak camera in conventional mode. A narrow slit of 40 μm wide in the horizontal direction (x_s in Supplementary Figure 13a) was inserted into the streak camera's entrance. By operating the streak camera at 0.5 THz, we acquired streak images that contain temporal evolutions of fluorescence decay. A single-component exponential fit was employed to extract lifetimes (Supplementary Figure 14d). Note that this measurement approach has been extensively applied in the literature [14-16]. Therefore, we adopted the results of this standard measurement as our references. The reconstructed lifetimes from CUSP are 498 ps, 310 ps, and 248 ps for the concentrations of 22 mM, 36 mM, and 40 mM, respectively. They match well with the reference lifetimes of 512 ps, 307 ps, and 235 ps. The errors are <15 ps, much less than the lifetimes. In addition, they both follow the lifetime-concentration trend previously demonstrated in the literature [11].

Fluorescence emission spectra

The fluorescence emission spectra reconstructed from passive CUSP and those measured by a spectrometer are plotted in Supplementary Figure 14d. These plots indicate that a higher fluorophore concentration shifts the emission peak slightly to longer wavelengths [13]. Note that the reconstructed spectra are slightly wider than the spectrometer measurements due to the convolution with the system's spectral PSF.

Supplementary Note 10: Characterizations of spatial resolutions

Spatial resolution is usually compromised in compressed sensing when a 2D image multiplexes information from more dimensions [7, 8, 17-22]. To experimentally characterize CUSP's spatial resolutions, we illuminated a custom-made photomask (Front Range Photomask) with a broadband pulse train generated by the beamsplitter pair (see Supplementary Figure 6). The imaging optics has a magnification of 1×. The photomask contains a spoke pattern, and its static image taken by the external camera is given in Supplementary Figure 15a. The spatial frequency response of this static image was calculated by 2D Fourier transformation. Then we imaged and

reconstructed this scene using both the active and the passive CUSP systems. The datasets from CUSP were analyzed by first integrating in the spectral and temporal domains to get 2D images and then Fourier transforming the 2D images. Compared to the static image, passive CUSP isotropically degrades the spatial resolution by 2.1×, while active CUSP experiences a 2.8× degradation (Supplementary Figures 15b-15d). Here, spatial resolution is defined as the noise-limited bandwidth at 2σ above the background, where σ is the standard deviation of the background noise [8].

Supplementary Table 1

Sparsity analysis of the multi-dimensional transient scenes imaged by CUSP. Sparsity is defined here as the number of voxels with intensity below a certain threshold (denoted as zero-valued voxel) divided by the total number of voxels in the reconstructed dataset [23]. We used the background mean plus the standard deviation of the background noise as the threshold.

Scene	System	Data density	Data sparsity
Chirped pulse train sweeping a pattern	Active CUSP	2.1%	97.9%
Pulse propagation in a Kerr medium (experiment 1)	Active CUSP	6.9%	93.1%
Pulse propagation in a Kerr medium (experiment 2)	Active CUSP	3.2%	96.8%
SR-FLIM (Rh6G 22 mM)	Passive CUSP	4.7%	95.3%
SR-FLIM (Rh6G 36 mM)	Passive CUSP	5.7%	94.3%
SR-FLIM (Rh6G 40 mM)	Passive CUSP	4.5%	95.5%

Supplementary Figures

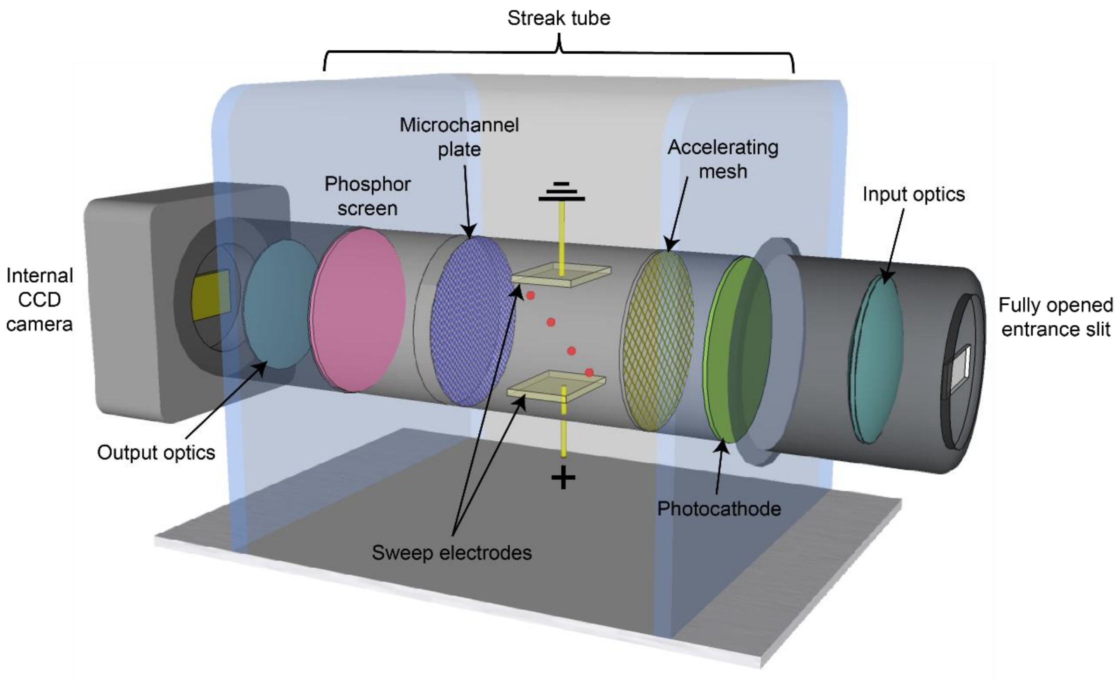

Supplementary Figure 1

Detailed illustration of a streak camera. Red dots between the sweep electrodes represent accelerated photoelectrons of different times of arrival. The top ones arrive earlier than the bottom ones. A sweeping voltage is applied to the sweep electrodes in streak mode, while no sweeping voltage is applied in focus mode.

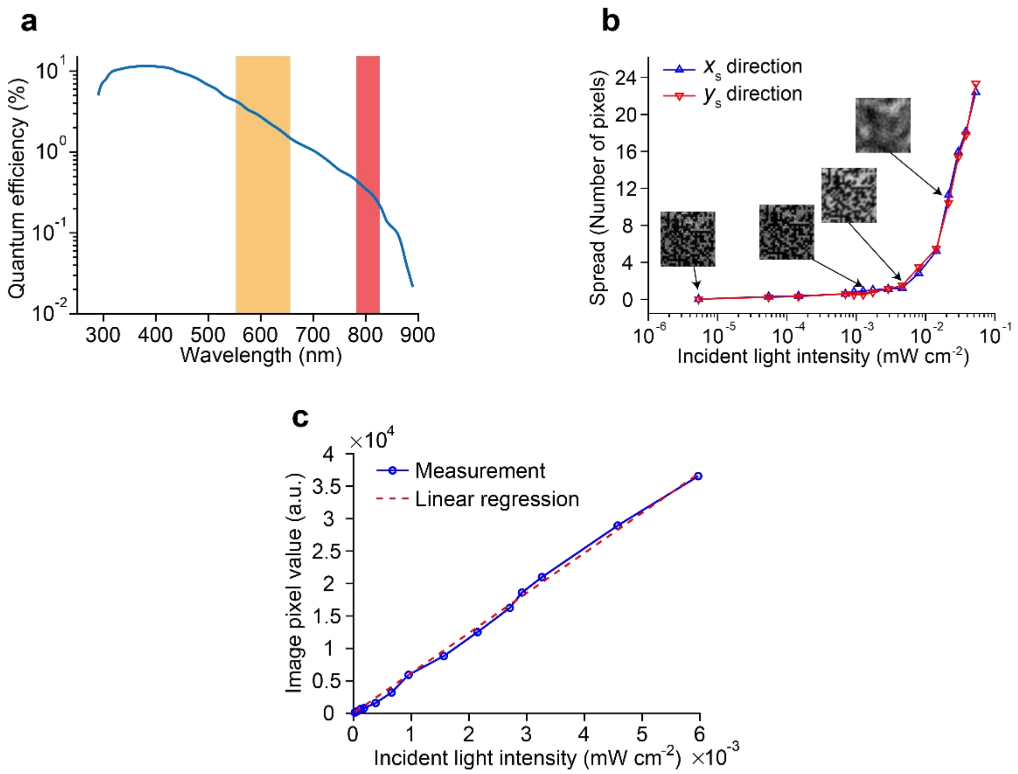

Supplementary Figure 2

Characterizations of the streak camera. (**a**) Measured quantum efficiency of the photocathode in the streak tube without the input optics. The orange and the red bands stand for the bandwidths used in SR-FLIM and 70-Tfps active imaging, respectively. (**b**) Space-charge induced image spread at different incident light intensities. Spread is expressed as the number of sensor pixels. Insets: two-dimensional images captured by the streak camera in focus mode at four selected light intensities. (**c**) Response curve of the streak camera.

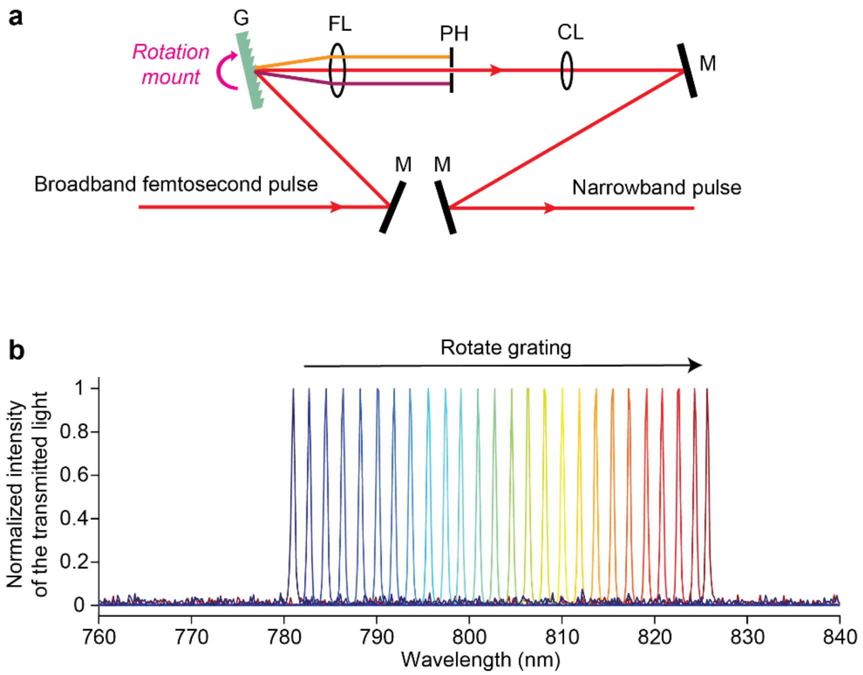

Supplementary Figure 3

Tunable bandpass filter system for characterizing the active CUSP system in the spectral domain. (**a**) Schematic of the system. CL, 100-mm-focal-length collimation lens (Thorlabs, LA1509); FL, 100-mm-focal-length focusing lens (Thorlabs, LA1050); G, 1200 lp mm^{-1} reflective diffraction grating (Thorlabs, GR25-1208); M, mirror; PH, 50-µm-diameter pinhole (Thorlabs, P50C). (**b**) Measured transmission spectra as the grating rotates.

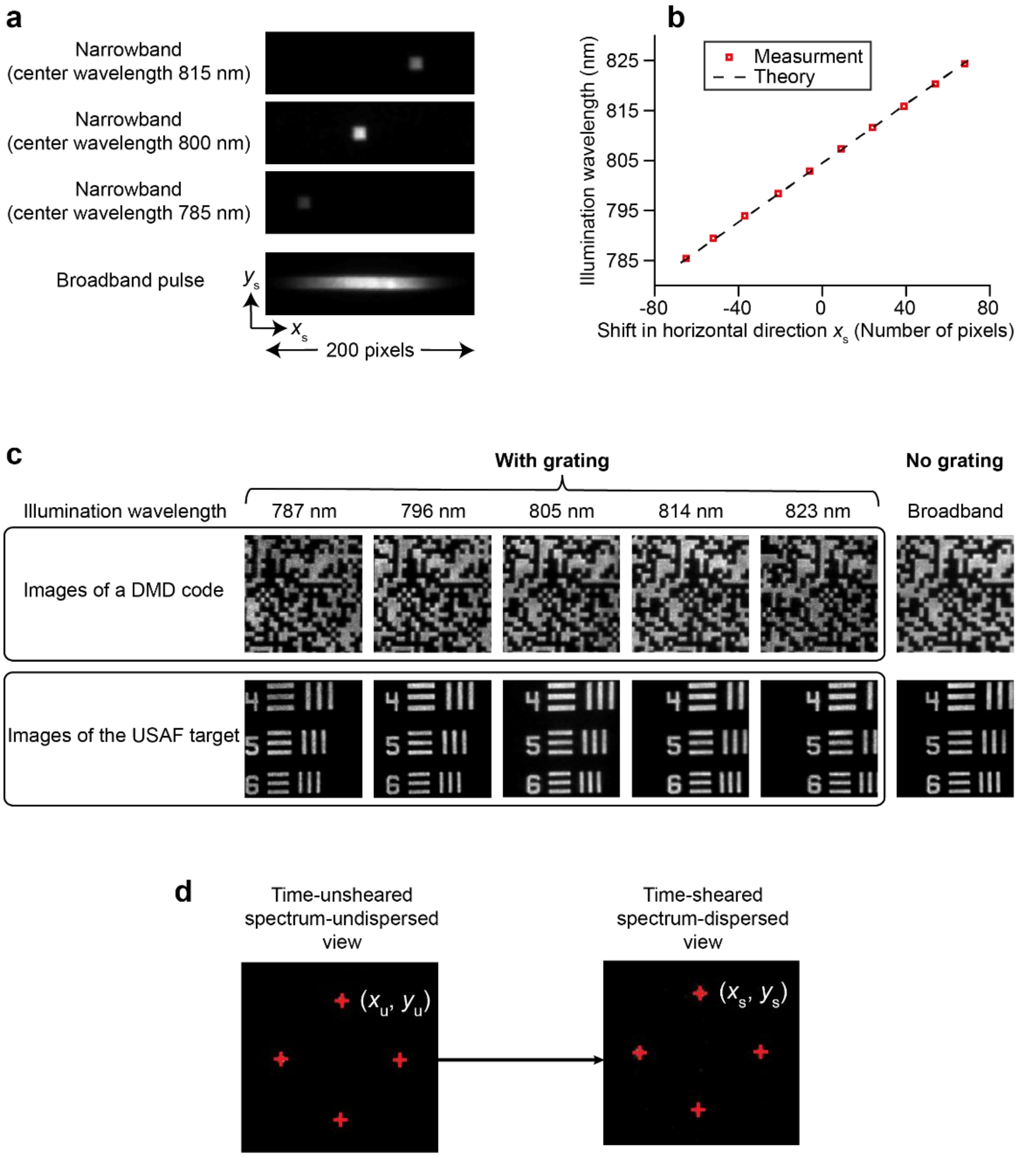

Supplementary Figure 4

Characterizations of the imaging section of the active CUSP system. (**a**) and (**b**) Dispersion characterization of the active CUSP system. (**a**) Images taken by the streak camera in focus mode when the object is illuminated by the filtered narrowband pulses (first three rows) and the broadband pulse (bottom row). (**b**) Wavelength of the narrowband illumination versus horizontal shift of the image from experiment (red squares) and theory (black dashed line). (**c**) Images taken by the streak camera for active CUSP at five illumination wavelengths, picked by the tunable bandpass filter. (**d**) Image distortion calibration.

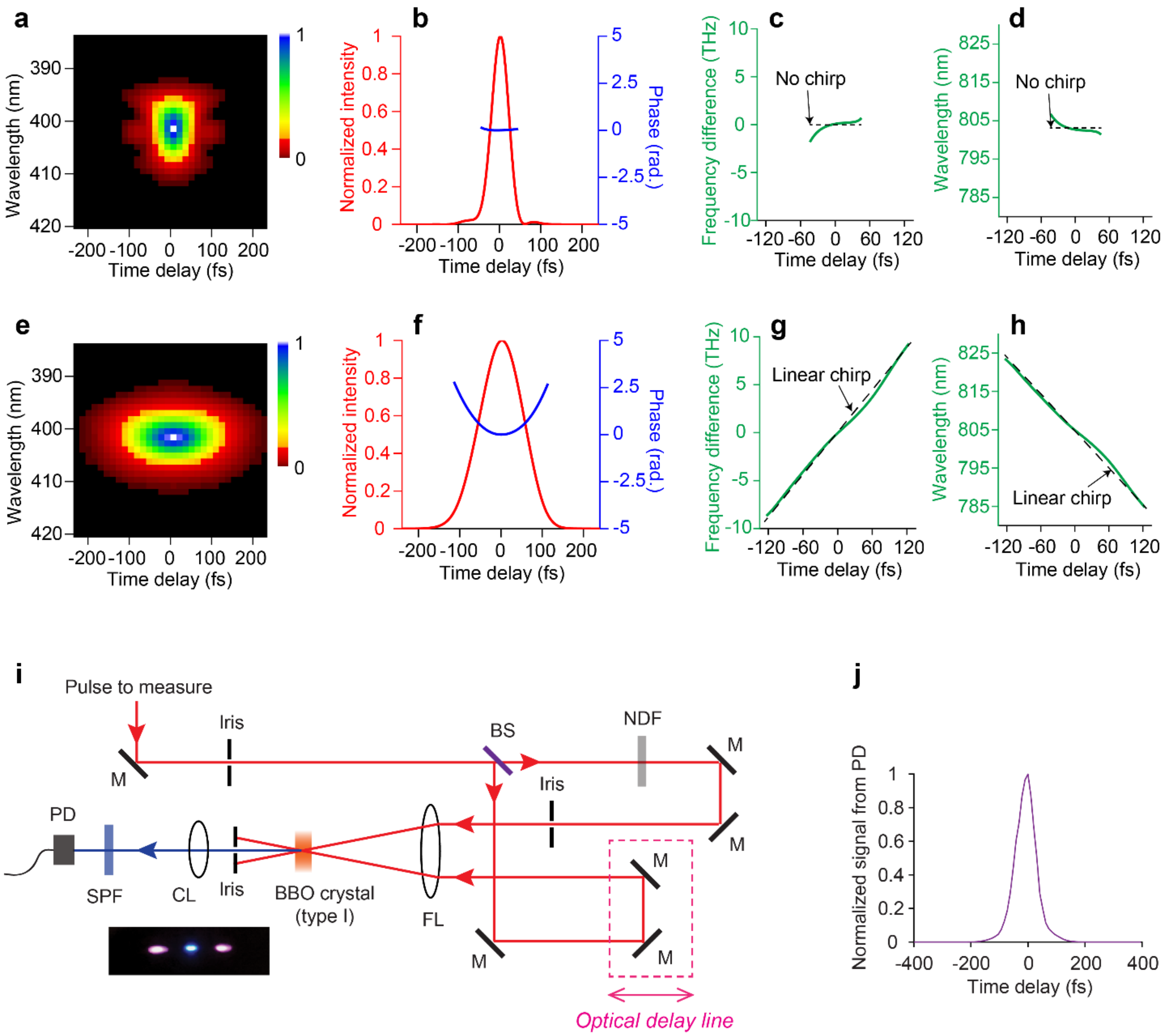

Supplementary Figure 5

Characterization of ultrashort optical pulses in the illumination section of the active CUSP system. FROG measurements of (**a**)-(**d**) the original femtosecond pulse, and (**e**)-(**h**) after propagating the entire CUSP imaging section. (**a**) and (**e**), retrieved FROG traces. (**b**) and (**f**), reconstructed pulse intensity and phase in the time domain. (**c**) and (**g**), calculated optical frequency difference versus time. (**d**) and (**h**), convert optical frequency in (**c**) and (**g**) to wavelength. In (**c**) and (**d**), black short-dashed lines represent a transform-limited pulse with no chirp. In (**g**) and (**h**), black long-dashed lines represent a pulse with perfectly linear chirp. (**i**) Schematic of the intensity auto-correlator for stretched pulse characterization. BS, 50/50 (R/T) non-polarizing plate beamsplitter (Thorlabs, EBS1); CL, 50-mm-focal-length collimation lens

(Thorlabs, LBF254-050-A); FL, 100-mm-focal-length focusing lens (Thorlabs, LA1050); M, mirror; NDF, neutral density filter (Thorlabs, NE05A) to equalize intensities; PD, photodiode (Thorlabs, DET36A); SPF, short-pass filter (Thorlabs, FESH0600). The optical delay line is controlled by a precision linear stage (PI, PLS-85). Inset: photograph of the SFG beam (center) and two fundamental beams after BBO. (**j**) Intensity auto-correlation measurement of a 48-fs Gaussian-shape pulse.

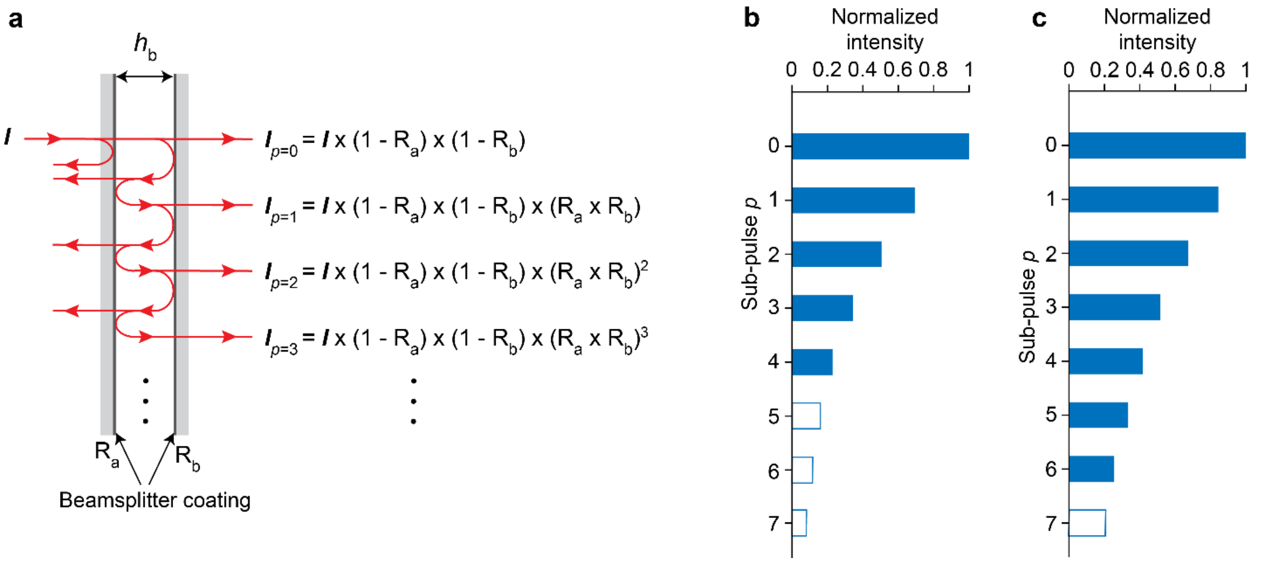

Supplementary Figure 6

Pulse train generator for 70-Tfps imaging. (**a**) Schematic of the dual beamsplitter setup. (**b**) Normalized intensities of five usable sub-pulses generated by a 90/10 (R/T) beamsplitter (Thorlabs, BSX11R) and a 70/30 (R/T) beamsplitter (Thorlabs, BST11R). (**c**) Normalized intensities of seven usable sub-pulses generated by two 90/10 (R/T) beamsplitters (Thorlabs, BSX11R). These two configurations are for the experiments shown in Fig. 2 and Fig. 3, respectively. Sub-pulses plotted as solid bars were used in the experiments, while those shown as hollow bars were discarded.

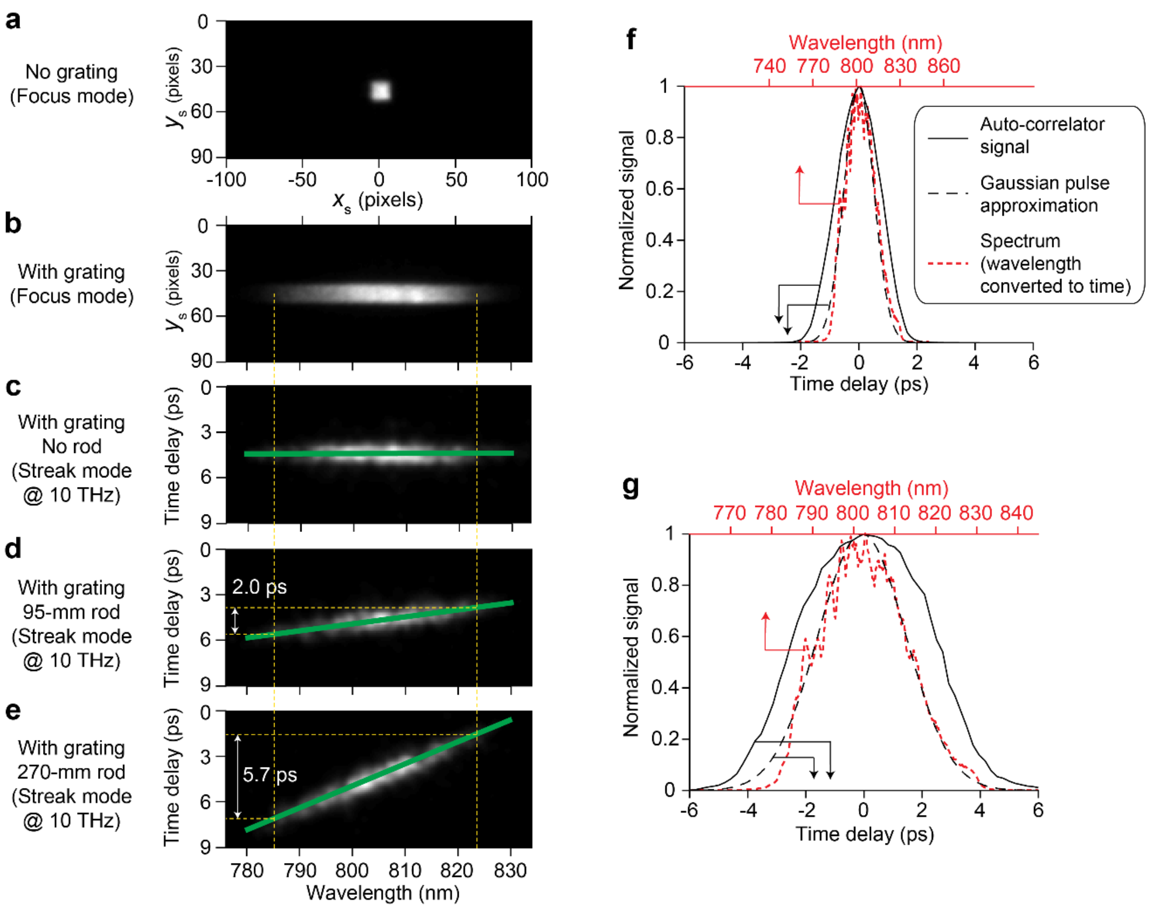

Supplementary Figure 7

Characterizing the pulses stretched by glass rods. (**a**) and (**b**) Images acquired by the streak camera in focus mode (**a**) without and (**b**) with the diffraction grating. (**c**)-(**e**) Images acquired by the streak camera in 10-THz streak mode when (**c**) no rod, (**d**) a 95-mm rod, and (**e**) a 270-mm rod is used. Yellow dashed lines delineate the bandwidth used in the 70-Tfps imaging experiments. Green solid lines are the theoretical estimations of linear temporal chirps by the glass rods. (**f**) and (**g**) Black solid lines are the auto-correlator measurements of the pulses stretched by (**f**) a 95-mm-long rod and (**g**) a 270-mm-long rod. Black long-dashed lines are Gaussian-pulse approximations based on the FWHMs of the auto-correlator signals. Broadband spectra resolved by a spectrometer are plotted in red short-dashed lines, after converting wavelength to time based on linear chirps.

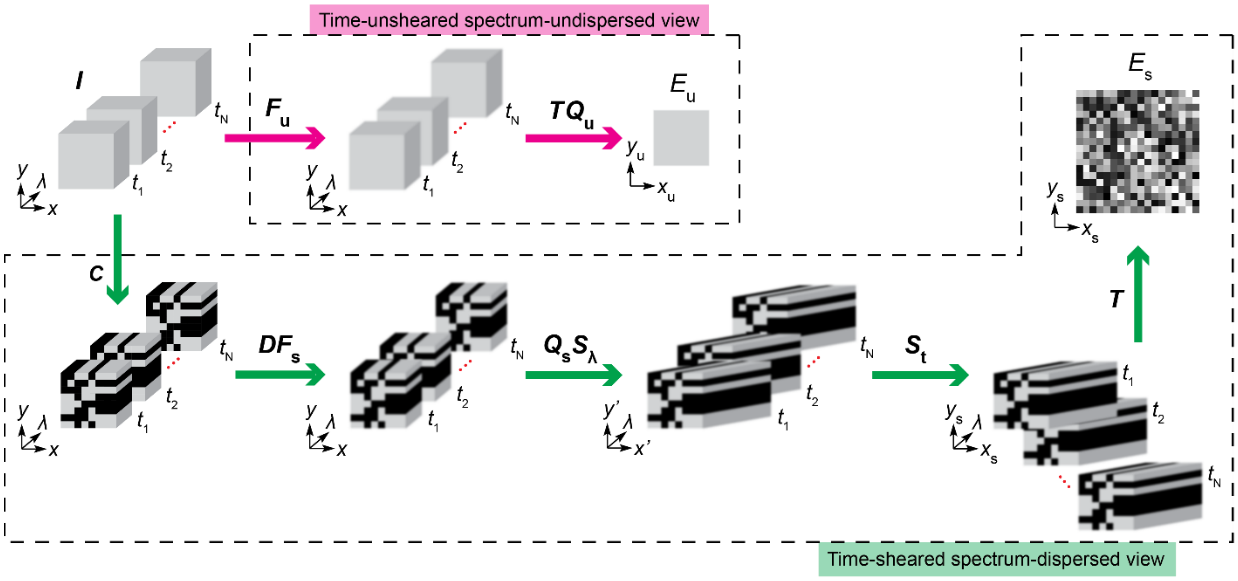

Supplementary Figure 8

Data acquisition process of CUSP. x, y, spatial coordinates of the transient scene; x_{u}, y_{u}, spatial coordinates of the external CCD camera; x', y', intermediate spatial coordinates at the entrance port of the streak camera; x_{s}, y_{s}, spatial coordinates of the streak camera; λ, wavelength; t, time; $\boldsymbol{C}$, spatial encoding operator; $\boldsymbol{F}_{\mathbf{u}}$, $\boldsymbol{F}_{\mathbf{s}}$, low-pass-filtering operators that blur the images; $\boldsymbol{D}$, image distortion operator of the streak camera with respect to the external CCD; $\boldsymbol{S}_{\boldsymbol{\lambda}}$, spectral dispersion operator by the diffraction grating; $\boldsymbol{S}_{\mathbf{t}}$, temporal shearing operator by the streak camera; $\boldsymbol{Q}_{\mathbf{u}}$, $\boldsymbol{Q}_{\mathbf{s}}$, spectral quantum efficiencies of the external CCD and the photocathode of the streak camera, respectively; $\boldsymbol{T}$, spatiotemporal-spectrotemporal integration operator; E_{u}, E_{s}, CCD images from *u*-View and *s*-View, respectively. Note that in order to visualize a 4D matrix in the x-y-t-λ space, we stack multiple 3D matrices in the x-y-λ space at different t values. Totally N frames in t space is assumed in this diagram.

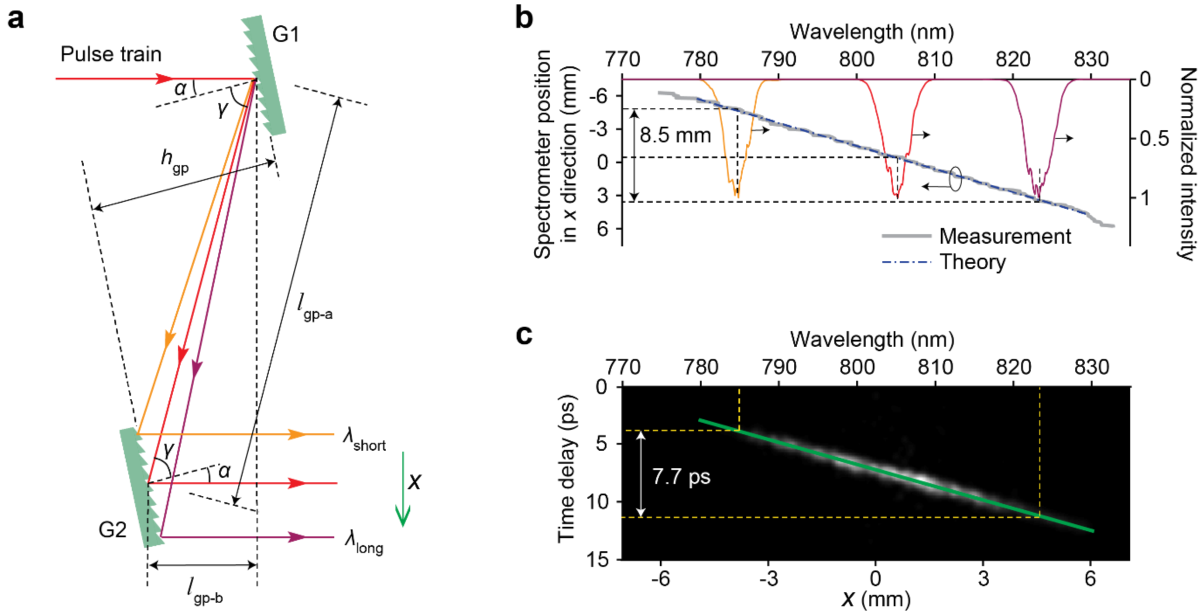

Supplementary Figure 9

Spatial and temporal chirps generated by a grating pair. (**a**) Schematic of the grating pair setup. (**b**) Spatial chirp, measured by a fiber-coupled spectrometer mounted on a translation stage. Gray solid line gives the peak wavelength of the measured spectrum as the spectrometer moves in the x direction. Blue dash-dotted line is from theory. Three exemplary spectrometer measurements are plotted in orange, red and purple solid lines. (**c**) Temporal chirp, measured by direct streak camera imaging at 10 THz. The object was a 1D narrow slit in the x direction. Theoretical estimation is plotted in green solid line. Within the bandwidth for imaging (785 nm to 823 nm), a positive chirp of 7.7 ps was measured.

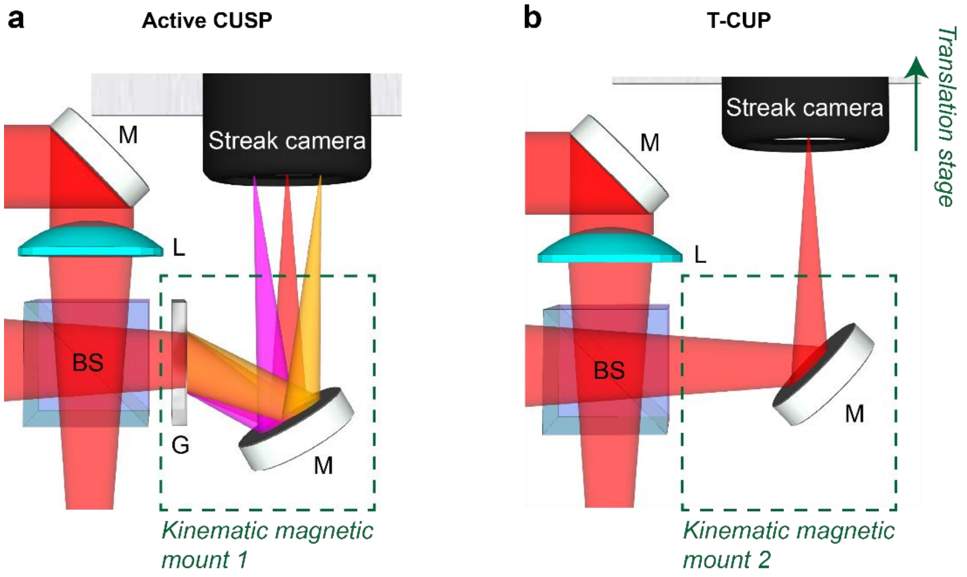

Supplementary Figure 10

Implementation of T-CUP. (**a**) Top view of the section of the active CUSP system near the streak camera's entrance (see the central inset of Fig. 1 in Main Text). (**b**) Top view of the same section for the implementation of T-CUP. The components enclosed in dashed boxes are mounted on two kinematic magnetic mounts (Thorlabs, KBT3X3) coupled with the same magnetic base (Thorlabs, KBB3X3) so that we can readily and repeatedly switch between CUSP and T-CUP. In addition, the streak camera has to be slightly shifted along the optical axis using a translation stage to accommodate T-CUP.

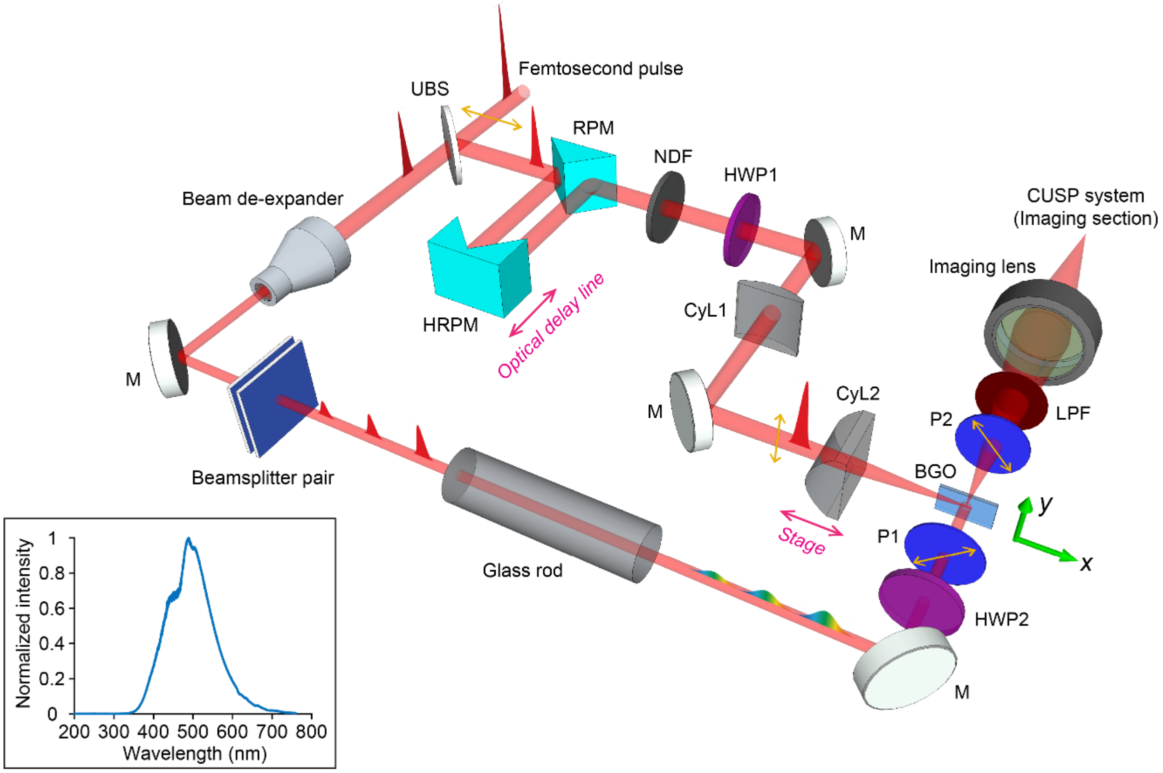

Supplementary Figure 11

Detailed schematic of the setup for imaging an ultrashort pulse propagation in a Kerr medium (BGO). Beam de-expander, (Thorlabs, LA1509-B, and Thorlabs, LC1439-B); CyL1, 300-mm-focal-length cylindrical lens (Thorlabs, LJ1558RM); CyL2, 25-mm-focal-length cylindrical lens (Thorlabs, LJ1075L2); HRPM, hollow-roof prism mirror (Thorlabs, HRS1015-P01); HWP1, half-wave-plate (Thorlabs, AHWP05M-980); HWP2, half-wave-plate (Thorlabs, WPH05M-780); LPF, long pass filter (Thorlabs, FGL715); M, mirror; NDF, neutral density filter (Thorlabs, NE05A); P1, linear polarizer (Thorlabs, LPVIS100-MP2); P2, linear polarizer (Newport, 05P109AR.16); RPM, right-angle prism mirror (Thorlabs, MRAK25-P01); UBS, ultrafast 50/50 (R/T) beamsplitter (Thorlabs, UFBS5050). The optical delay line is controlled by a precision stage (PI, PLS-85). Bottom left inset: Multi-photon-absorption induced fluorescence spectrum measured by a spectrometer.

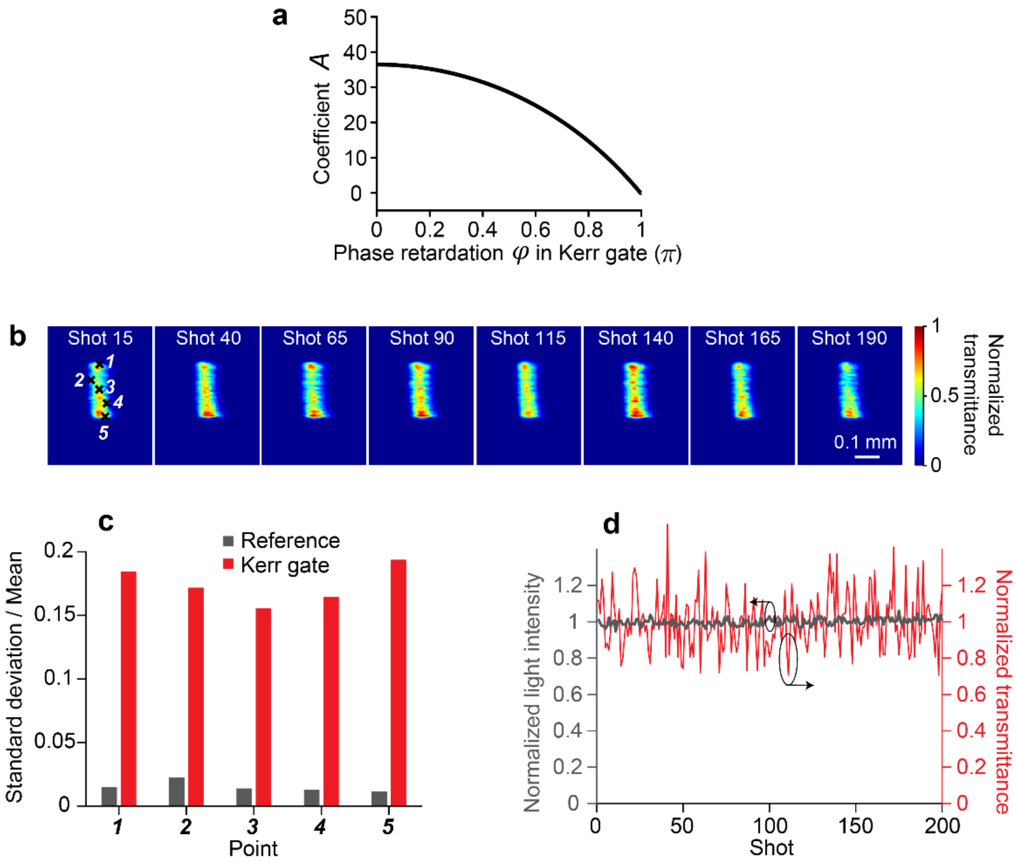

Supplementary Figure 12

Stability of the Kerr gate. (**a**) Coefficient *A* connecting the fractional change of the gate pulse intensity to that of the Kerr gate transmittance. (**b**)-(**d**) Experimental study on the stability of the Kerr gate. (**b**) Representative shots of the Kerr gate transmittance profile at a fixed time delay. (**c**) Relative change (standard deviation/mean) of the intensity from the reference experiment and that of the transmittance from the Kerr gate experiment, at five spatial points labelled in (**b**). (**d**) Sequences of intensity and transmittance from point 3. They are normalized to their mean values.

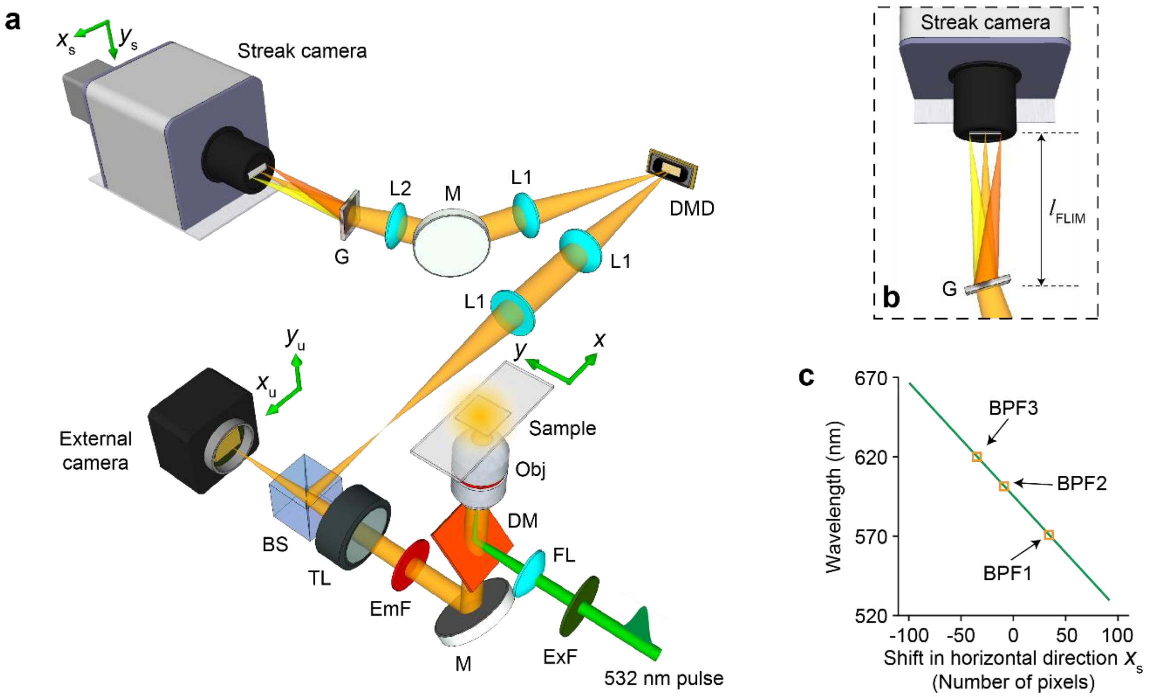

Supplementary Figure 13

Passive CUSP for SR-FLIM at 0.5 Tfps. (**a**) Schematic of the whole system. BS, 90/10 (R/T) beamsplitter (Thorlabs, BS028); DMD, digital micromirror device (Texas Instruments, LightCrafter 3000); G, 600 lp mm^{-1} transmission diffraction grating (Thorlabs, GT25-06V); L1, 150-mm-focal-length lens (Thorlabs, AC254-150-A); L2, 125-mm-focal-length lens (Thorlabs, AC254-125-A); M, mirror. Details of the other components can be found in Methods. (**b**) Zoom-in top-view of the system near the entrance of the streak camera. (**c**) Dispersion characterization of the passive CUSP system. The green solid line plots the theoretical calculation of the wavelength versus the horizontal spatial shift. The three orange squares represent the centers of the objects in the images when the fluorescence was filtered by three narrow bandpass filters, respectively. BPF1, BPF2, and BPF3, band-pass filters centered at 570 nm (Thorlabs, FB570-10), at 600 nm (Thorlabs, FB600-10), and at 620 nm (Thorlabs, FB620-10), respectively.

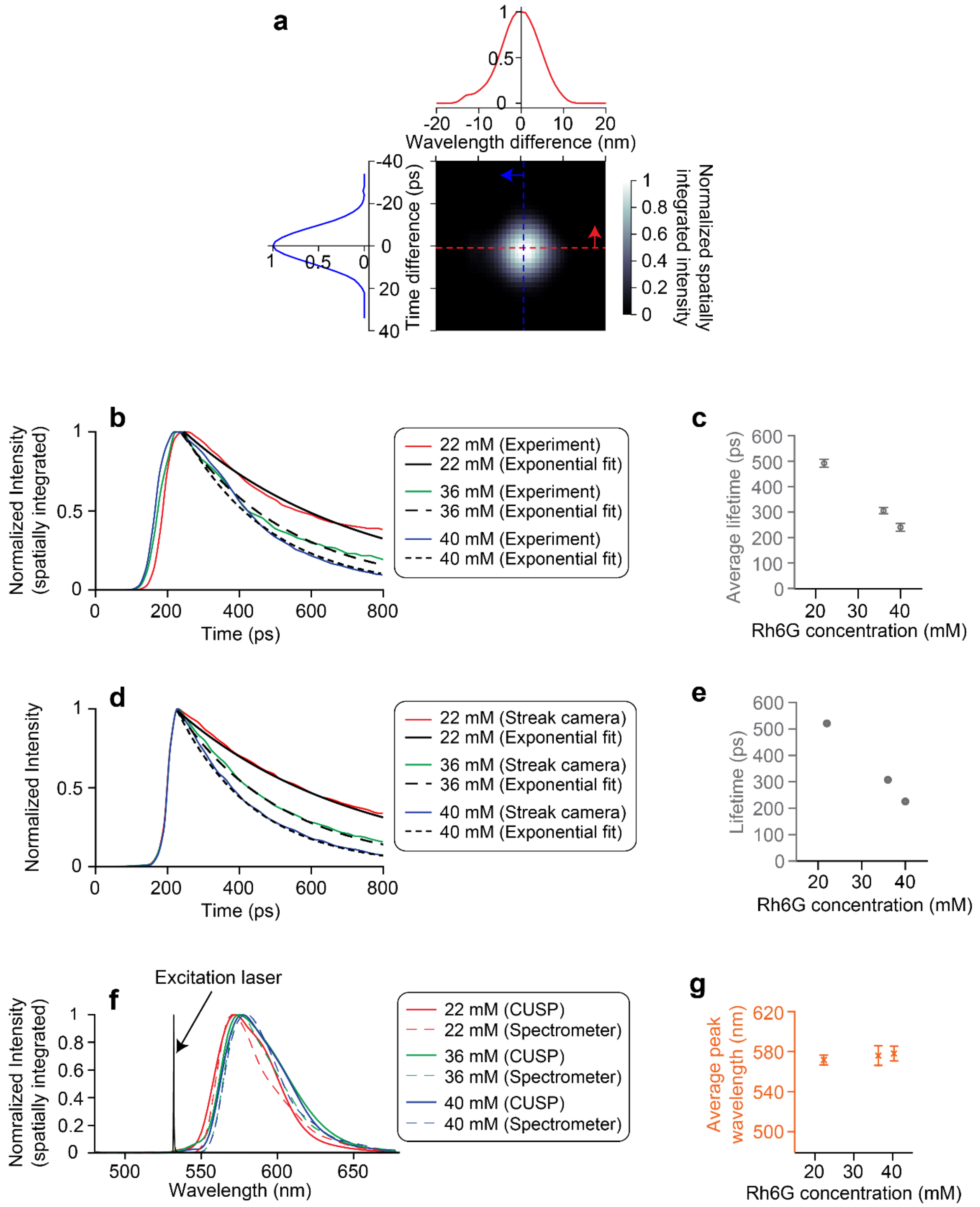

Supplementary Figure 14

Additional results for SR-FLIM. (**a**) 2D PSF in the t-λ space for the passive CUSP system. Blue and red solid lines on the left and top sides are cross-sections of the 2D PSF through its peak. (**b**) and (**c**) Fluorescence lifetimes. (**b**) Evolutions of the spatially integrated intensities at the peak

wavelengths of the three samples and their exponential fits. (**c**) Reconstructed average lifetimes for different fluorophore concentrations. (**d**) and (**e**) Reference fluorescence lifetimes measured by direct streak camera imaging. (**d**) Evolutions of the fluorescence emission intensities of the three samples and their exponential fits. (**e**) Measured reference lifetimes for different concentrations. (**f**) and (**g**) Spectra of fluorescence emissions. (**f**) Spatially integrated fluorescence emission spectra. Solid lines are from the passive CUSP reconstructions, and dashed lines are the reference measurements by a spectrometer. (**g**) Reconstructed average peak wavelengths of the emission spectra for different fluorophore concentrations. Error bars: standard deviations.

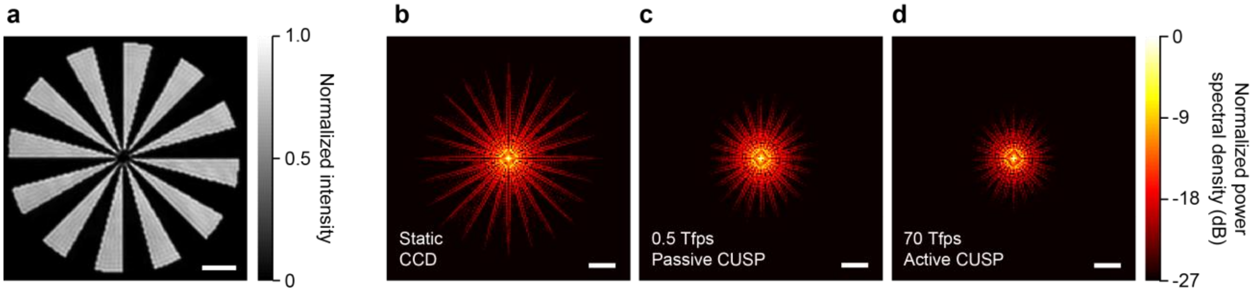

Supplementary Figure 15

Characterizations of CUSP's spatial resolutions. (**a**) Static image of a spoke pattern taken by the external CCD camera and then co-registered to the streak camera's view. Scale bar: 0.3 mm. (**b**)-(**d**) Spatial frequency responses of (**b**) the static image in (**a**), the spectrotemporally integrated images from (**c**) passive CUSP at 0.5 Tfps and (**d**) active CUSP at 70 Tfps. Scale bars in (**b**)-(**d**): 10 mm^{-1}.

Supplementary References

1. B. L. Qian and H. E. Elsayed-Ali, “Electron pulse broadening due to space charge effects in a photoelectron gun for electron diffraction and streak camera systems,” *Journal of Applied Physics* **91**, 462-468 (2001).
2. *Guide to Streak Cameras* https://www.hamamatsu.com/resources/pdf/sys/SHSS0006E_STREAK.pdf (Hamamatsu Corp., Hamamatsu City, Japan, 2008).
3. *Femtosecond streak camera FESCA-200 datasheet* https://www.electronicsdatasheets.com/manufacturers/hamamatsu/parts/c6138--fesca200- (Hamamatsu Corp., Hamamatsu City, Japan, 2008).
4. *What Is Camera Calibration?* https://www.mathworks.com/help/vision/ug/camera-calibration.html (The MathWorks, Inc.).
5. Z. Zhengyou, “Flexible camera calibration by viewing a plane from unknown orientations,” *Proceedings of the Seventh IEEE International Conference on Computer Vision* **1**, 666-673 (1999).
6. K. T. Tang, *Mathematical Methods for Engineers and Scientists 3: Fourier Analysis, Partial Differential Equations and Variational Methods* (Springer, 2006).
7. J. Liang, C. Ma, L. Zhu, Y. Chen, L. Gao, and L. V. Wang, “Single-shot real-time video recording of a photonic Mach cone induced by a scattered light pulse,” *Science Advances* **3**, e1601814 (2017).
8. J. Liang, L. Zhu, and L. V. Wang, “Single-shot real-time femtosecond imaging of temporal focusing,” *Light: Science & Applications* **7**, 42 (2018).
9. Z. Yu, L. Gundlach, and P. Piotrowiak, “Efficiency and temporal response of crystalline Kerr media in collinear optical Kerr gating,” *Optics Letters* **36**, 2904-2906 (2011).
10. Y. R. Shen, *The principles of nonlinear optics* (Wiley, 2002).
11. K. A. Selanger, J. Falnes, and T. Sikkeland, “Fluorescence lifetime studies of Rhodamine 6G in methanol,” *Journal of Physical Chemistry* **81**, 1960-1963 (1977).
12. J. R. Lakowicz, *Principles of Fluorescence Spectroscopy* (Springer US, 2006).
13. C. V. Bindhu, S. S. Harilal, G. K. Varier, R. C. Issac, V. P. N. Nampoori, and C. P. G. Vallabhan, “Measurement of the absolute fluorescence quantum yield of rhodamine B solution using a dual-beam thermal lens technique,” *Journal of Physics D: Applied Physics* **29**, 1074-1079 (1996).
14. A. G. Doukas, P. Y. Lu, and R. R. Alfano, “Fluorescence Relaxation Kinetics from Rhodopsin and Isorhodopsin,” *Biophysical Journal* **35**, 547-550 (1981).
15. R. V. Krishnan, H. Saitoh, H. Terada, V. E. Centonze, and B. Herman, “Development of a multiphoton fluorescence lifetime imaging microscopy system using a streak camera,” *Review of Scientific Instruments* **74**, 2714-2721 (2003).
16. J. L. Zhang, S. Sun, M. J. Burek, C. Dory, Y.-K. Tzeng, K. A. Fischer, Y. Kelaita, K. G. Lagoudakis, M. Radulaski, Z.-X. Shen, N. A. Melosh, S. Chu, M. Lončar, and J. Vučković, “Strongly Cavity-Enhanced Spontaneous Emission from Silicon-Vacancy Centers in Diamond,” *Nano Letters* **18**, 1360-1365 (2018).
17. L. Gao, J. Liang, C. Li, and L. V. Wang, “Single-shot compressed ultrafast photography at one hundred billion frames per second,” *Nature* **516**, 74-77 (2014).
18. J. Liang and L. V. Wang, “Single-shot ultrafast optical imaging,” *Optica* **5**, 1113-1127 (2018).

19. C. Yang, D. Qi, X. Wang, F. Cao, Y. He, W. Wen, T. Jia, J. Tian, Z. Sun, L. Gao, S. Zhang, and L. V. Wang, "Optimizing codes for compressed ultrafast photography by the genetic algorithm," *Optica* **5**, 147-151 (2018).
20. Y. Lu, T. T. W. Wong, F. Chen, and L. Wang, "Compressed ultrafast spectral-temporal photography," *Physics Review Letters* **122**, 193904 (2019).
21. P. Llull, X. Liao, X. Yuan, J. Yang, D. Kittle, L. Carin, G. Sapiro, and D. J. Brady, "Coded aperture compressive temporal imaging," *Optics Express* **21**, 10526-10545 (2013).
22. A. Wagadarikar, R. John, R. Willett, and D. Brady, "Single disperser design for coded aperture snapshot spectral imaging," *Applied Optics* **47**, B44-B51 (2008).
23. G. H. Golub and C. F. V. Loan, *Matrix Computations* (Johns Hopkins University Press, 2013).
