# Supplementary material for: Single-shot ultrafast imaging attaining 70 trillion frames per second
Source: Nat Commun. 2020 Apr 29;11:2091. doi: 10.1038/s41467-020-15745-4 (PMC7190645; doi:10.1038/s41467-020-15745-4)
Supplement: Supplementary file 3 — Description of Additional Supplementary Information [file 41467_2020_15745_MOESM3_ESM.pdf]

Description of Additional Supplementary Files

File Name: Supplementary Movie 1

Description: Ultrafast dynamics of a spatially and temporally chirped laser pulse train sweeping a pattern of letters. Top left: imaged by the state-of-the-art T-CUP system at 10 Tfps. Top right: imaged by the active CUSP system at 70 Tfps. The 2D colormap uses color and grayscale to represent illumination wavelength and light intensity, respectively. Bottom: evolutions of light intensity at one selected spatial point from both T-CUP (red dashed line and red circle) and CUSP (gray solid line and gray triangles).

File Name: Supplementary Movie 2

Description: Ultrafast dynamics of the Kerr effect induced by a focused ultrashort laser pulse propagating in a BGO slab, imaged by active CUSP at 70 Tfps. There are 980 frames in total. Top: the first experiment when the gate focus is outside the field of view. Bottom: the second experiment when the gate focus is inside the field of view. The gray dashed lines show the boundaries of the gate pulse along the y-axis. The locally normalized transmittance ($T_{\mathrm{norm-Loc}}$) of the Kerr gate at eight spatial points $p_{1:i}$ and $p_{2:i}$ $(i = 1,2,3,4)$ are plotted as well.

File Name: Supplementary Movie 3

Description: Ultrafast dynamics of a focused laser pulse propagating in the Kerr medium, acquired by the T-CUP system at 10 Tfps (top left), the pump-probe method at 70 Tfps (top middle), and the active CUSP system at 70 Tfps (top right). The CUSP data is a segment of Experiment 1, from 3.87 ps to 5.37 ps, in Supplementary Movie 2. The locally normalized transmittances ($T_{\mathrm{norm-Loc}}$) of the Kerr gate at a spatial point (labelled by the cyan cross), measured by the three methods, are plotted at the bottom.

File Name: Supplementary Movie 4

Description: Ultrafast dynamics of fluorescence decay (Rhodamine 6G in methanol) in the x-y-λ space, imaged by passive CUSP at 0.5 Tfps. The concentrations are 20 mM (left), 36 mM (middle), and 40 mM (right). Top: intensity dynamics in the x-y-λ space. Bottom: normalized total intensity I_{TS} integrated over the 2D space domain, plotted against wavelength and time. There are 400 frames and 100 wavelength samples in total.
